# Chronic itch and lichenification: a classic case of lichen simplex chronicus

**DOI:** 10.11604/pamj.2025.52.117.49121

**Published:** 2025-11-19

**Authors:** Pawan Banduji Itankar, Gaurav Rajendra Sawarkar

**Affiliations:** 1 Department of Rachana Sharir, Mahatma Gandhi Ayurved College Hospital and Research Centre, Datta Meghe Institute of Higher Education and Research (Deemed to be University), Salod (H), Wardha, Maharashtra, India.

**Keywords:** Hyperpigmentation, neurodermatitis, lichen planus

## Image in medicine

A 52-year-old male manual laborer from a humid, tropical region presented with persistent thickened, itchy patches on the nape of the neck for the past few months. The condition began as mild itching, which gradually worsened, especially at night and during periods of stress. He reported frequent scratching and rubbing, which led to thickened, leathery skin in the affected areas. There was no history of systemic illness, trauma, or any new topical or oral medication. He had tried over-the-counter antihistamines and emollients, with only temporary relief. Over time, the intense itching and chronic scratching caused significant discomfort and sleep disturbance. On clinical examination, well-demarcated, lichenified plaques with hyperpigmentation, accentuated skin markings, and mild excoriations were observed over the posterior neck, extensor surfaces of both legs, and ankles. No signs of active infection or systemic involvement were noted. Based on the history and clinical findings, a diagnosis of Lichen Simplex Chronicus (LSC) was made. LSC is a chronic, localized neurodermatitis characterized by thickened, hyperpigmented, lichenified plaques that result from repetitive scratching or rubbing. The condition presents with persistent pruritus, typically worse at night, leading to the development of thickened, leathery plaques with hyperpigmentation and exaggerated skin markings (lichenification). Commonly involved sites include the nape of the neck, scalp, forearms, legs, and genitalia. Lichen simplex chronicus is recurrent and relapsing in nature, often requiring long-term management to break the itch-scratch cycle and prevent recurrence.

**Figure 1:**
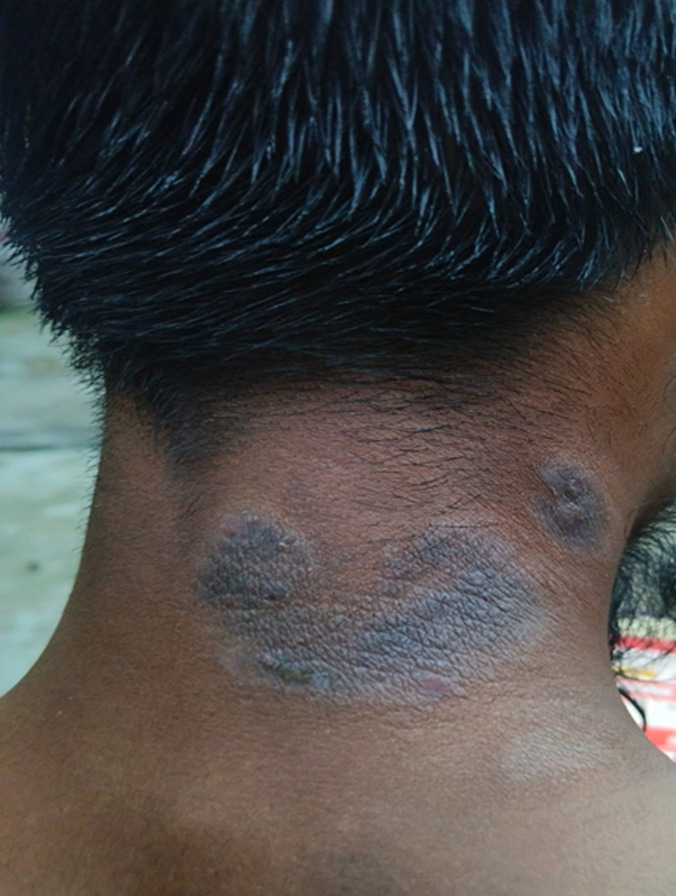
lichenified plaques with hyperpigmentation, exaggerated skin markings, and mild excoriations over nape of the neck

